# An online survey data in senior high school students and their parents in China during the outbreak of coronavirus disease 2019

**DOI:** 10.1016/j.dib.2022.108166

**Published:** 2022-04-14

**Authors:** Jian Feng Pei, Yeerzhati Yeerjiang, Hai Feng Gao, Lei Wang, Ruo Xin Zhang, Wang Hong Xu

**Affiliations:** a Department of Epidemiology, Fudan University School of Public Health, 138 Yi Xue Yuan Road, Shanghai 200032, China; b Admissions Office, Shanghai Medical College of Fudan University, 138 Yi Xue Yuan Road, Shanghai 200032, China

**Keywords:** Coronavirus disease 2019, Senior high school students, Attitude toward medical study, Anxiety symptom, Health literacy level on infectious diseases, COVID-19, Coronavirus disease 2019

## Abstract

The dataset presents the raw data collected through an online survey of senior high school students and their parents from 24 provinces, municipalities and autonomous regions (96 cities) of China. We conducted the online survey using electronic self-administered questionnaires designed as student-version and parent-version during 26th February and 4th March of 2020. The questionnaires were designed using the online survey tool Sojump (Shanghai Information Co.), and released through WeChat platform (Tencent Corp) following principals-head teachers-students/parents approach. All the students and the parents were asked to answer the questions voluntarily and anonymously after reading informed consent at the fore page of the questionnaires. The information collected from students included: 1) demographic characteristics, including sex, date of birth, name of high school, academic year, and self-evaluated performance level; 2) educational levels and occupations of parents; 3) degree preferences, including the willingness to learn medicine (prior and post COVID-19 outbreak), preferred medical career (clinician, public health practitioner, pharmacist, nurse or others), and main motivations for selecting or unselecting medical study; 4) infection of COVID-19 in acquaintances; 5) health literacy level on infectious diseases assessed using the Infectious Disease-specific Health Literacy Scale (IDSHL), and 6) anxiety level evaluated using the Chinese version of the Generalized Anxiety Disorder Screener (GAD-7). Information collected from parents included sex of their children and name of high school attended by their children, as well as their own educational level, occupation, anxiety symptoms, attitude toward their children's studying medicine, and main reasons for supportive or unsupportive attitudes, which were similar to the main motivations or de-motivations for medical study listed in the student-version questionnaire. Date and time for completion of the questionnaire were auto-recorded by the Sojump system. The dataset was established at the early stage of pandemic of COVID-19, and is valuable for understanding the instant psychological impacts of the outbreak of an emerging fatal infectious disease on senior high school students and their patents, and can provide evidence for policymakers on mental health intervention and medical education in China. The data are provided with this article.

## Specifications Table

**Table:** 

Subject	Epidemiology; Psychiatry and Mental Health; Medical Education
Specific subject area	Online survey in senior high school students and their parents during the outbreak of coronavirus disease 2019
Type of data	Categorical and numerical data presented in tables and figures; Excel and SAS files
How the data were acquired	Data were acquired through online survey of senior high school students and their parents using structured questionnaires in Chinese language. The English version of the questionnaires is provided as a supplementary file.
Data format	Raw, Analysed Dataset in Microsoft Excel (.xlsx) file format and in SAS file format
Description of data collection	The questionnaires were designed using the online survey tool Sojump (Shanghai Information Co.), and released through WeChat platform (Tencent Corp).
Data source location	Institution: Fudan University City/Town/Region: Shanghai Country: China
Data accessibility	Repository name: Mendeley Data, V1 Direct URL to data: https://data.mendeley.com/datasets/zpvfwnp9cp/6 DOI number: 10.17632/zpvfwnp9cp.6
Related research article	L. Wang, Y. Yeerjiang, H. F. Gao, J.F. Pei, R.X. Zhang, W.H.Xu, Self-reported anxiety level and related factors in senior high school students in China during the outbreak of coronavirus disease 2019, J. Affect Disord. 2022 [1]

## Value of the Data

• The dataset is important to understand the instant psychological impacts of an emerging fatal infectious disease and related measures fighting against the disease on Chinese senior high school students and their patents.
• The dataset may help to develop a novel health literacy intervention to reduce anxiety in Chinese senior high school students during the epidemic of COVID-19, for it provides scores for each domain of the Infectious Disease-specific Health Literacy Scale.
• The dataset may benefit medical educators, researchers and policymakers to improve current medical education systems as it offers a detailed account of senior high school students and their parents’ perception and concerns on medical study that could be used to enhance attractiveness of medical career in outstanding senior high school students.
• The dataset collected from students in key senior high schools may be used for a comparative analysis with those collected from general senior high schools. In addition, the data was collected at an early stage of pandemic of COVID-19, which may be used to evaluate the changes at later stages of epidemic.

## 1 Data Description

The outbreak of coronavirus disease 2019 (COVID-19) was first reported in December 2019 in Wuhan, China, and spread to all 34 provinces, municipalities and autonomous regions of the country very rapidly [2,3]. Extensive measures including travel restriction, social distancing, home confinement and coronavirus nucleic acid testing were taken to prevent the spread of the disease. People were asked to stay at home, and the opening of school was delayed indefinitely. The pandemic of COVID-19 affected people's life, leading to physically and mentally unhealthy [1]. Meanwhile, the efforts and achievements of Chinese medical staff in fighting against the fatal diseases gain them respect from the whole society, which may favour the choice of medical career in students [4].

The survey was conducted at an early stage of COVID-19 pandemic around the world when the first wave in China was initially under controlled. The English version of electronic questionnaires were available in the Mendeley Data as supplementary files of “Questionnaire (student).pdf” for students and “Questionnaire (parent).pdf” for parents. The final dataset comprised a total of 42,557 participants, with 21,141 students, 21,024 parent guardians and 392 non-parent guardians. Raw datasets were provided in the Mendeley Data as “student dataset raw.xlsx” and “parent dataset raw.xlsx”. The dataset of students was composed of 66 items which were defined as five types of variables including survey related information, socio-demographic and academic characteristics, preference to medical study, screen for general anxiety disorder, and assessment of infectious disease specific health literacy (IDSHL). The dataset of parents having 4 parts contains 38 items, for the IDSHL scale was not used in parents (Table 1). The description and assignment of each variable in the two datasets are clarified in Table 2. Online questionnaires can only be submitted after all the questions are answered, so there's no missing value in the collected data theoretically. However, “PROVINCE” values for 76 observations (56 students and 20 parents) can't be inferred from the school locations and were missing. Data cleaning was conducted using SAS code available in the Mendeley Data as “Data cleaning.sas” and “SAS code for Data cleaning.txt”. The origin question corresponding to each variable can be seen from the questionnaires attached to this article. Finally, the clean datasets were provided in the form of Excel named “student dataset cleaned.xls” and “parent dataset cleaned.xls” with rows representing observations and columns designating variables. All non-parent guardians were excluded from the analyses due to small sample size of the subjects and aiming to focus on parents only. Details about the datasets for 21,141 students and 21,024 parent guardians were described below.

**Table 1:** Type of variables in the student dataset and the parent dataset.

Type	Number of variables
Dataset for students	
Survey related information	4
Socio-demographic and academic characteristics	6
Preference to medical study	17
Screening for generalized anxiety disorder	7
Assessment of infectious disease specific health literacy	
Basic information	4
True or false questions	10
Multiple choice questions	13
Reading comprehension	5
Total	66
Dataset for parents	
Survey related information	4
Socio-demographic and academic characteristics	10
Preference to medical study for their children	17
Screening for generalized anxiety disorder	7
Total	38

**Table 2:** Description of variables in the student dataset and the parent dataset.

Variables	Description of variables	Levels	Available observations
Dataset for students			
ID	Identification number	-	21,141
DATE	Date of answering the questionnaire	-	21,141
TIME	Time at completing questionnaire	-	21,141
ETIME	Time spent on answering questionnaire	Continuous variable	21,141
GENDER	Gender of participants	1-Men; 2-Women	21,141
BIRTH_YEAR	Birth year of participants	-	21,141
FA_EDU	Educational level of father	1-Primary school or below; 2-Junior high school; 3-Senior high school or technical school; 4-Junior college; 5-College or above	21,141
MA_EDU	Educational level of mother	1-Primary school or below; 2-Junior high school; 3-Senior high school or technical school; 4-Junior college; 5-College or above	21,141
PROVINCE	Residence of participants	-	21,085
GRADE	Senior high school grades	1-Year one; 2-Year two; 3-Graduate year; 4-Resit of graduate year	21,141
ACADEMIC_PERFORMANCE	Estimated best colleges can be admitted according to academic performance	1-Top-tier; 2-Second tier; 3-Third tier; 4-Others	21,141
WILLINGNESS_BEFORE	Intention to study medicine before COVID-19	1-Yes; 2-No; 3-It does not matter	21,141
WILLINGNESS_NOW	Current intention to study medicine	1-Yes; 2-No; 3-It does not matter	21,141
MAIN_REASON	The main reason to study medicine	1-Interested in medicine; 2-Family expectation; 3-Great contribution to society; 4-Respected by others; 5-High salary; 6-Stable job; 7-Can help family members; 8-Others	6,264
UNWILL_1 to UNWILL_10	Reasons for unwilling to study medicine	1-Yes, it is my concern; 0-No, it is not my concern	11,151
EXPECTED_MAJOR	Which major want to study most	1-Clinical medicine; 2-Public health; 3-Pharmacy; 4-Nursing; 5-Chinese traditional medicine; 6-Chinese pharmacy; 7-Others	9,990
DECISION_MAKING	Power to make decision on career choosing	1-Absolutely not by myself, my parents will make the decision; 2-Probably not, it is mainly up to my parents; 3-Possible yes, my parents and I will make the decision together; 4-Probably yes, I will make the decision, and prefer to consult with my parents; 5-Absolutely by myself, I will make the decision	21,141
COVID19CASE	Anyone you know diagnosed with COVID-19	1-Yes; 2-No	21,141
GAD7_1 to GAD7_7	Seven items of the GAD-7	1-Not at all; 2-Several days; 3-More than half the days; 4-Nearly every day	21,141
PHY_CONDITION	Self-assessed physical condition	1-Very good; 2-Good; 3-Average; 4-Bad; 5-Very bad	21,141
MED	Visited a doctor, or suspended your work or schooling, or kept to your bed due to illness or injury during past 2 weeks	1-Yes; 2-No	21,141
OL_TIME	Average time for internet surfing every day	1-Never; 2-<1 hour; 3-1 to 2 hours;4-2 to 3 hours; 5->3 hours	21,141
INFO_ACCESS	Main approach to obtain knowledge or information on health	1-TV; 2-Internet; 3-Radio; 4-Newspaper or magazines; 5-Healthcare personnel; 6-Family members, colleagues or friends; 7-Others	21,141
TFQ1 to TFQ10	True or false questions of IDSHL questionnaire	1-Agree; 2-Disagree; 3-I don't know	21,141
MCQ1 to MCQ13	Multiple choice questions of IDSHL questionnaire	1 to 4 are correspond to the choices in each multiple-choice question	21,141
RC_1 to RC_5	Reading comprehension of IDSHL questionnaire	1 to 3 are correspond to the choices in each multiple-choice question	21,141
Dataset for parents			
ID	Identification number	-	21,416
DATE	Date of answering the questionnaire	-	21,416
TIME	Time at completing questionnaire	-	21,416
ETIME	Time spent on answering questionnaire	Continuous variable	21,416
GENDER	Gender of participants	1-Men; 2-Women	21,416
BIRTH_YEAR	Birth year of participants	-	21,416
EDU	Educational level of participants	1-No formal education; 2-Primary school; 3-Junior high school; 4-Senior high school; 5-Technical or secondary school; 6-Vocational high school; 7-Junior college; 8-College or above	21,416
MARRIAGE	Marital status	1-Married; 2-Devoiced; 3-Widowed; 4-Unmarried; 5-Others	21,416
OCCUPATION	Occupation	1-Adminitative personnel of government, enterprises or institutions; 2-Professional or technical personnel; 3-Staff or clerk; 4-Businessman; 5-Personnel in agriculture, forestry, animal husbandry, fishery or water conservancy; 6-Equipment operators for production or transportation; 7-Soldier; 8-Student; 9-Others	21,416
MED_PROF	Medical professional	1-Yes; 2-No	21,416
RELATIONSHIP	Relationship with the student surveyed	1-Parents; 2-Parental grandparents;3-Maternal grandparents; 4-Others	21,416
ONLY_CHILD	Whether your child surveyed is only child	1-Yes; 2-No	21,024
CHI_GENDER	Gender of your child surveyed	1-Men; 2-Women	21,416
PROVINCE	Residence of participants	-	21,396
GRADE	High school grade of your child	1-Year one; 2-Year two; 3-Graduate year; 4-Resit of graduate year	21,416
ACADEMIC_PERFORMANCE	Estimated best colleges can be admitted for your child surveyed according to his/her academic performance	1-Top-tier; 2-Second tier; 3-Third tier; 4-Junior college; 5-Others	21,416
WILLINGNESS_BEFORE	Intention for your child to study medicine before COVID-19	1-Yes; 2-No; 3-It does not matter	21,416
WILLINGNESS_NOW	Current intention for your child to study medicine	1-Yes; 2-No; 3-It does not matter	21,416
MAIN_REASON	Main reason for your expecting your child to study medicine	1-Great contribution to society; 2-Respected by others; 3-High salary; 4-Stable job; 5-Can help family members; 6-Interested in medicine; 7-Others	10,099
UNWILL_1 to UNWILL_9	Reasons for expecting your child not to study medicine	1-Yes, it is my concern; 0-No, it is not my concern	5,014
EXPECTED_MAJOR	If you expect your child to study medicine, which major do you want him/her to study most	1-Clinical medicine; 2-Public health; 3-Pharmacy; 4-Nursing; 5-Chinese traditional medicine; 6-Chinese pharmacy; 7-Others	16,402
DECISION_MAKING	Power to make decision on child's career choosing	1-absolutely by me, my child always follows our advice; 2-probably by me, my child may follow our advice; 3-possible by me, my child may consult with us before making a decision; 4-probably not by me, my child usually makes his/her own decision; 5-absolutely not by me, my child always makes his/her own decision	21,416
COVID19CASE	Anyone you know diagnosed with COVID-19	1-Yes; 2-No	21,416
GAD7_1 to GAD7_7	Seven items of the GAD-7	1-Not at all; 2-Several days; 3-More than half the days; 4-Nearly every day	21,416

**Fig. 1:**
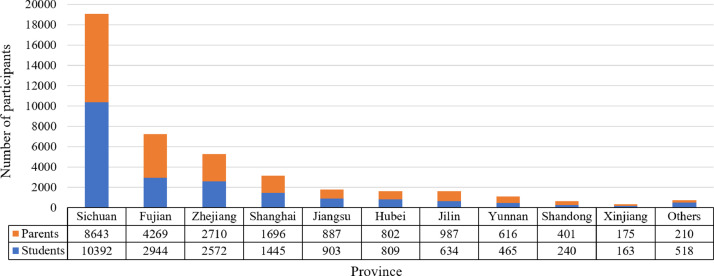
Geographic distribution of participants.

Geographic distribution of participants is displayed in Fig. 1. Province was determined by school locations. Regions with number of participants less than 200 were classified into “Others”. Details of socio-demographic and academic characteristics are showed in Table 3. Age of participants was calculated based on the year of survey and the year of birth. The students were more likely to be female. The number of students distributes equally from grade one to graduate year, all of which were larger than that of students at resit of graduate year. Most guardian participants were women, married, non-medical professionals, parents, and had only one child.

**Table 3:** Socio-demographic and academic characteristics of participants of the survey.

Variables	Mean/Frequency	SD/Percentage (%)
Dataset for students		
Age (years, mean, SD)	17.1	2.8
Gender		
Male	9,969	47.2
Female	11,172	52.8
Grade		
Year one	7,053	33.4
Year two	6,707	31.7
Graduate year	7,001	33.1
Resit of graduate year	380	1.8
Educational level of fathers		
Primary school or below	1,737	8.2
Junior high school	5,839	27.6
Senior high school or technical school	5,943	28.1
Junior college	2,979	14.1
College or above	4,643	22.0
Educational level of mothers		
Primary school or below	2,878	13.6
Junior high school	6,243	29.5
Senior high school or technical school	4,946	23.4
Junior college	3,343	15.8
College or above	3,731	17.7
Dataset for parents		
Age (years, mean, SD)	44.1	6.1
Gender		
Men	7,274	34.0
Women	14,141	66.0
Educational level		
No formal education	73	0.3
Primary school	1,206	5.6
Junior high school	4,675	21.8
Senior high school	2,626	12.3
Technical or secondary school	2,316	10.8
Vocational high school	243	1.1
Junior college	4,319	20.2
College or above	5,958	27.8
Marital status		
Married	20,089	93.8
Devoiced	919	4.3
Widowed	194	0.9
Unmarried	175	0.8
Others	39	0.2
Occupation		
Administrative personnel	4,189	19.6
Professional or technical personnel	3,684	17.2
Staff or clerk	2,633	12.3
Businessman	4,698	21.9
Personnel in agriculture, forestry, animal husbandry, fishery or water conservancy	620	2.9
Equipment operators for production or transportation	635	3.0
Soldier	79	0.4
Student	116	0.5
Others	4,762	22.2
Medical professional		
Yes	1,051	4.9
No	20,365	95.1
Relationship with the student		
Parents	21,024	98.2
Parental grandparents	37	0.2
Maternal grandparents	16	0.1
Others	339	1.6
Whether the student is your only child		
Yes	13,156	62.6
No	7,868	37.4
Gender of your child surveyed		
Male	10,983	51.3
Female	10,433	48.7
Grade of your child surveyed		
Year one	6,766	31.6
Year two	6,413	29.9
Graduate year	7,999	37.4
Resit of graduate year	238	1.11

SD: Standard Deviation

Table 4 shows the preference to medical study before and after the COVID-19 outbreak. Questionnaires of students and parents are similar in this part, including children's performance, students’ intention of studying medicine, expected medical majors, career decision makings. Only participant choose “Yes” in question “Now do you plan to apply for a medical school?” can answer question “If you plan to study medicine, what is the main reason for the plan”. When participant choose “No”, he/she should answer “If you have no plan to study medicine, what are your major concerns?”.

**Table 4:** Distribution of responses in relation to preference to medical study.

	Students			Parents*		
Questions and choices	Male (n=9,969)	Female (n=11,172)	Overall (n=21,141)	Fathers (n=7,156)	Mothers (n=13,868)	Overall (n=21,024)
According to your (child) academic performance, what do you think is the best college for you (your child)						
Top-tier	7,309 (73.3)	7,415 (66.4)	14,724 (69.7)	5,438 (76.0)	10,405 (75.7)	15,942 (75.8)
Second tier	1,887 (18.9)	2,807 (25.1)	4,694 (22.2)	1,329 (18.6)	2,579 (18.6)	3,908 (18.6)
Third tier	319 (3.2)	375 (3.4)	694 (3.3)	173 (2.4)	334 (2.4)	507 (2.4)
Junior college	-	-	-	111 (1.6)	217 (1.6)	328 (1.6)
Others	454 (4.6)	575 (5.2)	1,029 (4.9)	105 (1.5)	234 (1.7)	339 (1.6)
Intention of studying medicine before COVID-19						
Yes	1,593 (16.0)	2,103 (18.8)	3,696 (17.5)	2,700 (37.7)	5,096 (36.8)	7,796 (37.1)
No	6,950 (69.7)	7,791 (69.7)	14,741 (69.7)	2,099 (29.3)	4,500 (32.5)	6,599 (31.4)
It doesn't matter	1,426 (14.3)	1,278 (11.4)	2,704 (12.8)	2,357 (32.9)	4,272 (30.8)	6,629 (31.5)
Intention of studying medicine now						
Yes	2,669 (26.8)	3,595 (32.2)	6,264 (29.6)	3,489 (48.8)	6,444 (46.5)	9,933 (47.3)
No	5,366 (53.8)	5,785 (51.8)	11,151 (52.8)	1,469 (20.5)	3,451 (24.9)	4,920 (23.4)
It doesn't matter	1,934 (19.4)	1,792 (16.0)	3,726 (17.6)	2,198 (30.7)	3,973 (28.7)	6,171 (29.4)
Main reason for planning to apply for medical school						
Interested in medicine	866 (32.5)	1,325 (36.9)	2,191 (35.0)	442 (12.7)	886 (13.8)	1,328 (13.4)
Family expectation	138 (5.2)	135 (3.8)	273 (4.4)	-	-	-
Great contribution to society	1,129 (42.3)	1,440 (40.1)	2,569 (41.0)	2,349 (67.3)	4,121 (64.0)	6,470 (65.1)
Respected by others	63 (2.4)	38 (1.1)	101 (1.6)	180 (5.2)	278 (4.3)	458 (4.6)
High salary	70 (2.6)	46 (1.3)	116 (1.9)	20 (0.6)	32 (0.5)	52 (0.5)
Stable job	114 (4.3)	117 (3.3)	231 (3.7)	322 (9.2)	609 (9.5)	931 (9.4)
Can help family members	200 (7.5)	370 (10.3)	570 (9.1)	138 (4.0)	393 (6.1)	531 (5.4)
Others	89 (3.3)	124 (3.5)	213 (3.4)	38 (1.1)	125 (1.9)	163 (1.6)
Concerns for not planning to study medicine						
Not interested in medicine	4,402 (82.0)	4,307 (74.5)	8,709 (78.1)	1,168 (79.5)	2,803 (81.2)	3,971 (80.7)
No support from family	273 (5.1)	366 (6.3)	639 (5.7)	-	-	-
Heavy workload	874 (16.3)	1,082 (18.7)	1,956 (17.5)	264 (18.0)	681 (19.7)	945 (19.2)
High responsibility	760 (14.2)	880 (15.2)	1,640 (14.7)	228 (15.5)	701 (20.3)	929 (18.9)
High risk of infection	391 (7.3)	375 (6.5)	766 (6.9)	137 (9.3)	407 (11.8)	544 (11.1)
Violence against doctors	903 (16.8)	1,165 (20.1)	2,068 (18.6)	239 (16.3)	651 (18.9)	890 (18.1)
Low salary	225 (4.2)	182 (3.2)	407 (3.7)	60 (4.1)	134 (3.9)	194 (3.9)
Crowded working environment	325 (6.1)	281 (4.9)	606 (5.4)	79 (5.4)	190 (5.5)	269 (5.5)
Long training	1,010 (18.8)	928 (16.0)	1,938 (17.4)	150 (10.2)	319 (9.2)	469 (9.5)
Others	658 (12.3)	1,181 (20.4)	1,839 (16.5)	110 (7.5)	222 (6.4)	332 (6.8)
Which kind of medical major want to apply for most						
Clinical medicine	2,624 (57.0)	2,823 (52.4)	5,447 (54.5)	3,107 (54.6)	5,356 (51.4)	8,463 (52.6)
Public health	216 (4.7)	157 (2.9)	373 (3.7)	457 (8.0)	514 (4.9)	971 (6.0)
Pharmacy	382 (8.3)	305 (5.7)	687 (6.9)	224 (3.9)	465 (4.5)	689 (4.3)
Nursing	41 (0.9)	217 (4.0)	258 (2.6)	73 (1.3)	166 (1.6)	239 (1.5)
Chinese traditional medicine	548 (11.9)	856 (15.9)	1,404 (14.1)	1,114 (19.6)	2,344 (22.5)	3,458 (21.5)
Chinese pharmacy	181 (3.9)	380 (7.1)	561 (5.6)	187 (3.3)	408 (3.9)	595 (3.7)
Others	611 (13.3)	649 (12.1)	1,260 (12.6)	525 (9.2)	1,164 (11.2)	1,689 (10.5)
Can you decide what career you pursuit						
Parents make the decision	60 (0.6)	44 (0.4)	104 (0.5)	31 (0.4)	46 (0.3)	77 (0.4)
Mainly up to parents	369 (3.7)	360 (3.2)	729 (3.5)	329 (4.6)	424 (3.1)	753 (3.6)
Make the decision together	3,835 (38.5)	4,541 (40.7)	8,376 (39.6)	3,638 (50.8)	7,137 (51.5)	10,775 (51.3)
Prefer to consult with parents	5,088 (51.0)	5,876 (52.6)	10,964 (51.9)	2,477 (34.6)	4,960 (35.8)	7,437 (35.4)
All by children	617 (6.2)	351 (3.1)	968 (4.6)	681 (9.5)	1,301 (9.4)	1,982 (9.4)
Is there any one you know infected with COVID-19						
Yes	174 (1.8)	158 (1.4)	332 (1.6)	125 (1.8)	266 (1.9)	391 (1.9)
No	9,795 (98.3)	11,014 (98.6)	20,809 (98.4)	7,031 (98.3)	13,602 (98.1)	20,633 (98.1)

⁎ 392 non-parent guardians excluded.

The anxiety symptoms were assessed in students and parents respectively using the Chinese version of the Generalized Anxiety Disorder Screener (GAD-7) [5,6]. The score of the GAD-7 was used to classify anxiety levels, with score of 0 to 4 as minimal, 5 to 9 as mild, 10 to 14 as moderate, and 15 to 21 as severe anxiety. Table 5 presents distribution of responses in relation to GAD-7 scale in students and parents by sex.

**Table 5:** Distribution of responses in relation to GAD-7 scale.

	Students			Parents⁎⁎		
Items of GAD-7*	Male (n=9,969)	Female (n=11,172)	Overall (n=21,141)	Fathers (n=7,156)	Mothers (n=13,868)	Overall (n=21,024)
1. Feeling nervous, anxious, or on edge?						
Not at all	6,217 (62.4)	5,597 (50.1)	11,814 (55.9)	4,916 (68.7)	8,587 (61.9)	13,503 (64.2)
Several days	2,924 (29.3)	4,396 (39.4)	7,320 (34.6)	1,722 (24.1)	4,040 (29.1)	5,762 (27.4)
More than half the days	523 (5.3)	751 (6.7)	1,274 (6.0)	338 (4.7)	802 (5.8)	1,140 (5.4)
Nearly every day	305 (3.1)	428 (3.8)	733 (3.5)	180 (2.5)	439 (3.2)	619 (2.9)
2. Being unable to stop or control worrying?						
Not at all	7,671 (77.0)	7,684 (68.8)	15,355 (72.6)	5,758 (80.5)	10,707 (77.2)	16,465 (78.3)
Several days	1,765 (17.7)	2,706 (24.2)	4,471 (21.2)	1,012 (14.1)	2,253 (16.3)	3,265 (15.5)
More than half the days	340 (3.4)	501 (4.5)	841 (4.0)	217 (3.0)	530 (3.8)	747 (3.6)
Nearly every day	193 (1.9)	281 (2.5)	474 (2.2)	169 (2.4)	378 (2.7)	547 (2.6)
3. Worrying too much about different things?						
Not at all	6,994 (69.7)	7,068 (63.3)	14,012 (66.3)	5,115 (71.5)	9,329 (67.3)	14,444 (68.7)
Several days	2,213 (22.2)	3,055 (27.4)	5,268 (24.9)	1,525 (21.3)	3,311 (23.9)	4,836 (23.0)
More than half the days	528 (5.3)	671 (6.0)	1,199 (5.7)	326 (4.6)	762 (5.5)	1,088 (5.2)
Nearly every day	284 (2.9)	378 (3.4)	662 (3.1)	190 (2.7)	466 (3.4)	656 (3.1)
4. Having trouble relaxing?						
Not at all	7,578 (76.0)	8,033 (71.9)	15,611 (73.8)	5,984 (83.6)	11,250 (81.1)	17,234 (82.0)
Several days	1,779 (17.9)	2,382 (21.3)	4,161 (19.7)	830 (11.6)	1,826 (13.2)	2,656 (12.6)
More than half the days	382 (3.8)	492 (4.4)	874 (4.1)	196 (2.7)	475 (3.4)	671 (3.2)
Nearly every day	230 (2.3)	265 (2.4)	495 (2.3)	146 (2.0)	317 (2.3)	463 (2.2)
5. Being so restless that it is hard to sit still?						
Not at all	8,133 (81.6)	8,967 (80.3)	17,100 (80.9)	6,407 (89.5)	12,527 (90.3)	18,934 (90.1)
Several days	1,368 (13.8)	1,719 (15.4)	3,087 (14.6)	548 (7.7)	966 (7.0)	1,514 (7.2)
More than half the days	273 (2.7)	312 (2.8)	585 (2.8)	105 (1.5)	232 (1.7)	337 (1.6)
Nearly every day	195 (2.0)	174 (1.6)	369 (1.8)	96 (1.3)	143 (1.0)	239 (1.1)
6. Becoming easily annoyed or irritable?						
Not at all	6,986 (70.1)	7,203 (64.5)	14,189 (67.1)	5,935 (82.9)	11,008 (79.4)	16,943 (80.6)
Several days	2,171 (21.8)	2,868 (25.7)	5,039 (23.8)	950 (13.3)	2,264 (16.3)	3,214 (15.3)
More than half the days	511 (5.1)	704 (6.3)	1215 (5.8)	170 (2.4)	388 (2.8)	558 (2.7)
Nearly every day	301 (3.0)	397 (3.6)	698 (3.3)	101 (1.4)	208 (1.5)	309 (1.5)
7. Feeling afraid as if something awful might happen?						
Not at all	7,706 (77.3)	8,184 (73.3)	15,890 (75.2)	6,004 (83.9)	11,516 (83.0)	17,520 (83.3)
Several days	1,714 (17.2)	2,350 (21.0)	4,064 (19.2)	890 (12.4)	1,843 (13.3)	2,733 (13.0)
More than half the days	336 (3.4)	423 (3.8)	759 (3.6)	144 (2.0)	309 (2.2)	453 (2.2)
Nearly every day	213 (2.1)	215 (1.9)	428 (2.0)	118 (1.7)	200 (1.4)	318 (1.5)

⁎ GAD: Generalized anxiety disorder.

⁎⁎ 392 non-parent guardians excluded.

The IDSHL of students were measured using the scale developed by Tian et al [7]. The scale includes 4 subscales which are designed to measure basic knowledge, prevention, management or treatment of infectious diseases, and identification of pathogens and infection source. Supplementary Table 1 shows the correct answer to each question and the scoring of the IDSHL, while Table 6 presents distribution of responses in relation to IDSHL in student participants. The total score of IDSHL ranges from 0 to 100, with a higher score representing a higher level of health literacy. Most students reported with good or very good physical condition, didn't get illness or injury during past 2 weeks, spent more than 3 hours online. And the main approach for them to acquire health related information was internet*.*

**Table 6:** Distribution of responses in relation to infectious disease specific health literacy in student participants.

Variables/scores	Male students (n=9,969)	Female students (n=11,172)	Overall (n=21,141)
Physical condition			
Very good	5,711 (57.3)	5,317 (47.6)	11,028 (52.2)
Good	3,034 (30.4)	4,003 (35.8)	7,037 (33.3)
Average	1,159 (11.6)	1,779 (15.9)	2,938 (13.9)
Bad	47 (0.5)	70 (0.6)	117 (0.6)
Very bad	18 (0.2)	3 (0.0)	21 (0.1)
Illness or injury during past 2 weeks			
Yes	288 (2.9)	329 (2.9)	617 (2.9)
No	9,681 (97.1)	10,843 (97.1)	20,524 (97.1)
Average time spend online every day			
Never	217 (2.2)	141 (1.3)	358 (1.7)
<1 hour	910 (9.1)	801 (7.2)	1,711 (8.1)
1-2 hours	1,989 (20.0)	1,875 (16.8)	3,864 (18.3)
2-3 hours	1,482 (14.9)	1,589 (14.2)	3,071 (14.5)
>3 hours	5,371 (53.9)	6,766 (60.6)	12,137 (57.4)
Main approach to acquire health information			
TV	1,038 (10.4)	912 (8.2)	1,950 (9.2)
Internet	7,789 (78.1)	9,056 (81.1)	16,845 (79.7)
Radio	43 (0.4)	49 (0.4)	92 (0.4)
Newspaper or magazines	81 (0.8)	66 (0.6)	147 (0.7)
Healthcare personnel	572 (5.7)	698 (6.3)	1,270 (6.0)
Family members, colleagues or friends	111 (1.1)	125 (1.1)	236 (1.1)
Others	335 (3.4)	266 (2.4)	601 (2.8)
IDSHL score (mean ± SD)*	70.3 ± 16.9	71.6 ± 14.7	71.0 ± 15.8
Infectious disease-related knowledge and values	21.6 ± 7.2	22.5 ± 6.8	22.1 ± 7.0
Infectious disease prevention	20.7 ± 5.3	21.0 ± 4.6	20.9 ± 5.0
Management or treatment of infectious diseases	12.4 ± 6.5	12.4 ± 6.2	12.4 ± 6.4
Identification of pathogens and infection sources	15.5 ± 4.9	15.6 ± 4.6	15.6 ± 4.8

⁎ IDSHL: infectious disease specific health literacy.

## 2 Experimental Design, Materials and Methods

This cross-sectional study was conducted among senior high school students and their parents from 24 provinces, municipalities and autonomous regions (96 cities) of China between 26th February and 4th March in 2020 in China. The online questionnaires were designed as a student-version and a parent-version in the Sojump platform (Shanghai Information Co) and were released through WeChat platform (Tencent Corp) through convenience sampling approach. Specifically, the online questionnaires were forwarded to the principals of several key senior high schools by the Admissions Office of the Shanghai Medical College of Fudan University, and then to the head teachers of classes by the principals. Finally, the head teachers released the students’ and parents’ version of questionnaires to the corresponding WeChat groups consisting of students or guardian (mainly parents) only. In the parents’ group, only one parent was included for each student. In rare cases when neither of the parents was available, one grandparent or other family members would be included as the guardian of the student. All the students and the parents were asked to answer the questions voluntarily and anonymously after reading informed consent at the fore page of the questionnaires.

Duplicate questionnaires were removed based on IP address to ensure only one questionnaire was completed by one person. The principals and the head teachers were also encouraged to forward the online questionnaires to their colleagues in other key senior high schools.

## Ethics Statements

This study was approved by the Institutional Review Board of the Fudan University School of Public Health (IRB00002408 & FWA00002399). Informed consent was presented at the fore page of questionnaire. Once clicking the “start” button, the participant was assumed to have read the information about the survey, and voluntarily agree to participate in the study. Considering the nature of an anonymous online survey, and that senior high school students in China are usually at age of 16 or above, we did not seek for written consent form from legal guardians of student participants. However, the legal guardians (mainly parents) of all student subjects were informed of the survey in corresponding parent WeChat groups by head teachers and agreed their children's participation of the survey. The authors assert that all procedures contributing to this work comply with the ethical standards of the relevant national and institutional committees on human experimentation, the Helsinki Declaration of 1975, as revised in 2000, and the platform(s)’ data redistribution policies.

## CRediT authorship contribution statement

**Jian Feng Pei:** Data curation, Writing – original draft. **Yeerzhati Yeerjiang:** Visualization, Investigation. **Hai Feng Gao:** Supervision, Resources. **Lei Wang:** Software, Validation. **Ruo Xin Zhang:** Writing – review & editing. **Wang Hong Xu:** Conceptualization, Methodology, Funding acquisition, Software, Writing – review & editing.

## Declaration of Competing Interest

The authors declare that they have no known competing financial interests or personal relationships that could have appeared to influence the work reported in this paper.

## Data Availability

Effect of COVID-19 outbreak on generalized anxiety disorder and medical career decision of senior high school students in China (Original data) (Mendeley Data).

## Appendix Supplementary materials

Image, application 1
