# Supplementary material for: An online survey data in senior high school students and their parents in China during the outbreak of coronavirus disease 2019
Source: Data Brief. 2022 Apr 14;42:108166. doi: 10.1016/j.dib.2022.108166 (PMC9007934; doi:10.1016/j.dib.2022.108166)
Supplement: Supplementary file 1 [file mmc1.docx]

**Supplementary table 1 Difficulty coefficient and scores of infectious disease specific health literacy scale**

| **Question** | **Correct answer** | **Difficulty coefficient** | **Score** |
| --- | --- | --- | --- |
| **Part 1 Infectious diseases-related knowledge and values** | | |  |
| TFQ_3 | 1 | 4.08 | 10.57 |
| TFQ_5 | 2 | 1.19 | 3.08 |
| TFQ_6 | 2 | 1.41 | 3.65 |
| TFQ_7 | 2 | 1.22 | 3.16 |
| TFQ_8 | 2 | 1.21 | 3.13 |
| TFQ_9 | 2 | 1.34 | 3.47 |
| MCQ_10 | 1 | 2.72 | 7.04 |
| **Part 2 Prevention of infectious disease** | |  |  |
| MCQ_2 | 2 | 1.19 | 3.08 |
| MCQ_5 | 1 | 1.89 | 4.90 |
| MCQ_7 | 2 | 1.32 | 3.42 |
| MCQ_8 | 1 | 1.39 | 3.60 |
| MCQ_11 | 2 | 1.34 | 3.47 |
| MCQ_12 | 2 | 1.36 | 3.52 |
| MCQ_13 | 1 | 1.47 | 3.81 |
| **Part 3 Management or treatment of infectious diseases** | | | |
| TFQ_1 | 2 | 2.39 | 6.19 |
| TFQ_2 | 2 | 1.96 | 5.08 |
| TFQ_4 | 2 | 1.57 | 4.07 |
| TFQ_10 | 2 | 1.89 | 4.90 |
| **Part 4 Identification of pathogens and infection sources** | | |  |
| MCQ_1 | 3 | 1.88 | 4.87 |
| MCQ_4 | 2 | 2.38 | 6.16 |
| MCQ_6 | 2 | 1.97 | 5.1 |
| MCQ_9 | 3 | 1.45 | 3.76 |
